# Cost‐effectiveness of neuropsychological rehabilitation for acquired brain injuries: Update of Stolwyk et al.'s (2019) review

**DOI:** 10.1111/jnp.12387

**Published:** 2024-08-08

**Authors:** Mauro Mancuso, Ilaria Valentini, Michele Basile, Audrey Bowen, Helena Fordell, Roberta Laurita, Marika C. Möller, Lindy J. Williams, Pierluigi Zoccolotti

**Affiliations:** ^1^ Physical and Rehabilitative Medicine Unit NHS‐USL South‐East Tuscany Grosseto Italy; ^2^ Tuscany Rehabilitation Clinic, Montevarchi (AR) Montevarchi Italy; ^3^ Alta Scuola di Economia e Management dei Sistemi Sanitari (ALTEMS) – Università Cattolica del Sacro Cuore Rome Italy; ^4^ Manchester Centre for Health Psychology and Geoffrey Jefferson Brain Research Centre University of Manchester Manchester UK; ^5^ Department of Clinical Science, Neurosciences Umeå University Umeå Sweden; ^6^ Division of Rehabilitation Medicine, Department of Clinical Sciences, Danderyd Hospital Karolinska Institute Stockholm Sweden; ^7^ Department of Rehabilitation Medicine Danderyd University Hospital Stockholm Sweden; ^8^ Faculty of Health, School of Health Sciences Charles Darwin University Darwin Northern Territory Australia; ^9^ Department of Psychology Sapienza University of Rome Rome Italy

**Keywords:** acquired brain injury, cost‐effectiveness, neuropsychology, rehabilitation

## Abstract

Acquired brain injuries (ABI), resulting from stroke or traumatic brain injury, cause a range of neuropsychological impairments and many patients continue to experience neuropsychological deficits years after onset. The increasing average age of the population highlights the importance of effective management strategies for the consequences of ABI. Despite the well‐documented impact of rehabilitation interventions, the cost‐effectiveness of neuropsychological rehabilitation remains largely unknown. This study conducted a scoping review to update the findings of Stolwyk et al. (*Neuropsychological Rehabilitation*, 2021, 31, 316), focusing on the economic evaluations of neuropsychological rehabilitation for individuals with ABI. Following the PIO framework, PRISMA ScR guidelines, and systematic review reporting checklist, the review screened 1027 articles and included eight studies published between 2019 and 2024. The studies encompassed either language rehabilitation or general neuropsychological programs, including neuropsychological interventions. The economic analyses, including two cost‐effectiveness, five cost‐utility, and one cost–benefit study, mostly adhered to CHEERS guidelines, enhancing the transparency and methodological rigour of their reporting. These studies demonstrated varying degrees of cost‐effectiveness for interventions targeting post‐stroke language disorders and neuropsychological rehabilitation for ABI, with significant cost savings and health benefits observed, particularly for home‐based rehabilitation interventions. The included studies suffered from a short time horizon, limiting the ability to capture the long‐term economic impacts and effectiveness of the interventions. Future research should focus on longer‐term follow‐up data and include broader search strategies to enhance understanding and optimise health care interventions. A comprehensive implementation of these economic analyses is crucial for informing policymakers, enabling them to introduce rehabilitative interventions based on solid evidence.

## INTRODUCTION

Acquired brain injuries (ABI), such as those resulting from stroke or traumatic brain injury (TBI), produce a wide range of neuropsychological impairments, spanning cognitive, emotional, and behavioural domains. For example, cognitive impairments due to ABI affect 60%–90% of most individuals, as well as their family members and societies, as a whole (Lo et al., 2019; Mancuso et al., 2018). Recovery often requires a long time, and many patients are left with neuropsychological deficits several years after onset. Depending on the aetiology and severity, a substantial portion of patients experience permanent functional implications, severely affecting social participation and quality of life (Dams‐O'Connor et al., 2023; Gorgoraptis et al., 2019; Haarbauer‐Krupa et al., 2021). The progressive growth in the average age of the population (World Health Organization, 2015) increases the relevance of these disorders and the importance of adopting effective strategies for the management of the chronic consequences of acquired brain injuries. The population's ageing and the increased number of stroke survivors is estimated to be 27% more by the year 2047; therefore, rehabilitation is considered one of the most important health strategies in the 21st century (Stucki et al., 2018; Wafa et al., 2020; World Health Organization, 2017). Although the impact of these injuries on the lives of those affected is well documented, and many rehabilitation strategies have been proposed, the cost‐effectiveness of neuropsychological rehabilitation is largely unknown. To justify allocating health resources, national health services require evidence of interventions' cost and economic sustainability, such as those aimed at improving the quality of life of affected patients and their informal caregivers.

The brain injuries' impact varies considerably depending on the sites of injury and its aetiology, notwithstanding the influence of psychological coping and associated mental health problems. This symptomatologic complexity is reflected in the wide variety of rehabilitative interventions to improve the individual's neuropsychological functioning and adjust to persisting difficulties. Thus, some treatments focus solely on specific commonly occurring cognitive impairments, such as aphasia, spatial neglect, or memory difficulties. Others holistically address the patient's functional recovery, encompassing cognition, emotion, and social behaviour (Prigatano, 2019). In recent years, several literature reviews have examined the effectiveness of these rehabilitative interventions. Again, the picture that emerges is complex. Various reviews emphasise the efficacy of some interventions on selective deficits after brain injury (Cicerone et al., 2019; Rogers et al., 2018) or underscore the improvement of cognitive functioning and daily living based on computer‐assisted cognitive rehabilitation (e.g., Nie et al., 2022). There is also evidence that comprehensive holistic rehabilitation programs may improve psychosocial functioning in adults with behavioural and psychosocial disorders (Cattelani et al., 2010) and that cognitive behaviour therapy may reduce emotional distress after acquired brain injury (Bradbury et al., 2008). Understanding the needed intensity conditions and sufficient doses of cognitive training to reach a restorative effect in the brain and functioning in daily life, is still incomplete (e.g., Rogers et al., 2018). Furthermore, knowledge of the neural changes occurring with rehabilitation is still limited, although this goal has recently attracted considerable interest (e.g., Farokhi‐Sisakht et al., 2019). Thus, some evidence indicates that cognitive training may enhance resting‐state neural activity and connectivity, increasing the blood supply to these regions via neurovascular coupling (e.g., Chapman et al., 2015). Finally, one should also add that various reviews point out the limitations of the available literature concerning the methodological characteristics of neuropsychological rehabilitation studies, emphasising the need for future studies with high‐quality methodological designs (e.g., Allida et al., 2023; Longley et al., 2021, 2023).

Overall, neuropsychological rehabilitation is an important aid in the recovery of cognitive, emotional, and behavioural difficulties in individuals who have suffered a brain injury and can enable recovery, even if partial, by facilitating the individual's reintegration into daily life and, where possible, into the world of work. It therefore becomes important to have an effective assessment of the costs associated with neuropsychological rehabilitation so that the economic sustainability of these interventions can be evaluated, especially within public health systems. However, to date, a comprehensive evaluation of the sustainability of neuropsychological rehabilitation has proven difficult. Probably weighing on the difficulty of this assessment is the wide variety of sequelae related to brain injury that require differentiated and at least partially individualised interventions. Another complexity arises from the need to place the rehabilitation interventions within societal, institutional, and individual perspectives (i.e., an *analytical framework* in the terminology proposed by Solvang et al., 2016).

As an effect, information on the cost‐effectiveness of different neuropsychological interventions is limited. Stolwyk et al. (2021) performed a scoping review of the relevant literature up to 2019 and noted that several studies carried out a cost analysis of neuropsychological rehabilitation; however, only a few (8 studies) performed a cost‐effectiveness analysis with a control group. Understandably, the results were scattered as they referred to various interventions, and only three involved cognitive rehabilitation interventions. This body of evidence made it difficult to draw general conclusions. However, the authors also noted an interesting emerging tendency with recent clinical trials more prone to include economic evaluations.

This consideration prompted our interest in further examining the literature published after the scoping review by Stolwyk et al. (2021) that considered studies published between 1995 and May 2019. In the present review, we examined the studies from this latter date to the present (May 2024). For the sake of comparability, we used the same search terms and databases as in the original paper.

## METHODS

### Study design

We conducted an updated addition to the scoping review published by Stolwyk et al. (2021) to identify new studies focusing on the economic evaluation of neuropsychological rehabilitation or cognitive training interventions either as part of a single or multidisciplinary approach for individuals' post‐stroke or with brain injuries.

The review followed the PIO framework (Population, Intervention, and Outcome) (Mezaoui et al., 2019), the PRISMA ScR (PRISMA extension for Scoping Reviews) statement and guidelines (see Table S1) and the checklist for reporting systematic reviews (Tricco et al., 2018). In this study, the authors decided to use the PIO model (Mezaoui et al., 2019), omitting the inclusion of the comparator (the ‘C’) in the PICO model (McKenzie et al., 2023). This decision was made to broaden the search string and increase the likelihood of capturing all relevant studies, thereby minimising the risk of missing significant data.

### Eligibility criteria

Eligibility criteria were established following the PIO framework (Mezaoui et al., 2019). Studies from which data were extracted included published comprehensive economic evaluations conducted on post‐stroke or brain‐injured adult individuals in the context of clinical trials or model‐based studies examining treatments for neuropsychological rehabilitation in adults. The PIO criteria used in this review are displayed in Table 1.

**TABLE 1:** PIO criteria (Mezaoui et al., 2019).

Criteria	Description
Population	Adult individuals' post‐stroke or with traumatic brain injuries (Acquired Brain Injury)
Intervention	Neuropsychological rehabilitation or cognitive training interventions
Outcome	Economic evaluation such as cost‐effectiveness analysis, cost‐utility analysis, and cost–benefit analysis

As defined by Drummond (2015), cost‐effectiveness analysis (CEA) compares the costs and health outcomes of different interventions using a common measure, such as life years (LY) gained or blood pressure reduction. The output of CEA is the incremental cost per natural unit of consequence gained, expressed as the incremental cost‐effectiveness ratio (ICER). The Cost‐utility analysis (CUA) goes a step further by summarising the impacts on health‐related quality of life. These impacts are valued as ‘utilities’, such as quality‐adjusted life years (QALYs). The output of CUA is the incremental cost‐utility ratio (ICUR), which expresses the additional cost required to gain one additional QALY (Drummond, 2015), whereas the cost–benefit analysis (CBA) provides a broader perspective by summarising health and non‐health benefits in monetary terms, such as dollars. The result of CBA is typically expressed as net monetary benefits (INB) or cost–benefit ratio, allowing a direct comparison between the financial value of the benefits and the intervention costs (Drummond, 2015).

### Search strategy

The same search string as in the paper by Stolwyk et al. (2021) was used: (neuropsychology OR ‘cognitive intervention’ OR rehabilitation) AND (‘acquired brain injury’ OR ‘traumatic brain injury’ OR ‘brain injury’ OR ‘stroke’) AND ALL = (‘health economics’ OR ‘cost‐effectiveness’ OR ‘cost‐analysis’ OR ‘cost–benefit’) for the search period from 2019 to May 2024.

The search was conducted on the PubMed, Scopus, and Web of Science databases. Three reviewers (IV, RL, and MB) independently screened the articles for the title, abstract and full text. Disagreements were resolved by collectively revisiting the screening criteria. A fourth reviewer was consulted (MM) for unresolved discrepancies.

### Exclusion criteria and outcomes

Exclusion criteria included studies lacking cost data relevant to the target population, and non‐English language studies due to interpreter unavailability. Additionally, papers included by Stolwyk et al. (2021), conference abstracts, protocol studies, reviews, unpublished or grey literature, posters, and studies lacking full‐text availability, were excluded because this is an update to Stolwyk's work rather than a duplication.

The outcome measures for the economic review include ICER, ICUR, and INB per quality‐adjusted life year (QALY). QALYs quantify the value of health outcomes by combining life expectancy with a measure of quality of life (York Health Economics Consortium, 2016). One QALY equates to one year in perfect health. For example, if an individual lives for a year with a health condition that reduces their quality of life by half, they would accrue .5 QALYs. QALYs are often used in health economic evaluations to assess the effectiveness and efficiency of different medical treatments allowing the comparison of the relative benefits and costs of interventions across various diseases and conditions (York Health Economics Consortium, 2016).

### Data extraction

Data extraction was conducted using three separate tables. The first two tables included general study information such as author names and publication year, country, study participants (pathology), type of economic analysis (e.g., cost‐effectiveness, cost‐utility, or cost–benefit), costs, health outcome measures, horizon, interventions, and comparators, along with descriptions of them. The third table synthesised the economic information and study results, including study perspective, type and source(s) of cost data, mean costs, health effects (and if available, 95% CI), difference in incremental cost‐effectiveness ratio, and author's conclusion.

To facilitate international cost comparison the costs of interventions in each country were converted to euros (€) using the gross domestic product purchasing power parity, following the Organisation for Economic Co‐operation and Development recommendations, with Europe as the reference. According to the World Health Organization (WHO), a willingness‐to‐pay (WTP) threshold represents the amount a health care consumer might be willing to pay for a health benefit, typically based on a country's per capita gross domestic product (Bertram et al., 2016). The ICER and ICUR will be discussed in relationship to the Eurozone threshold, which amounts to €30,000–50,000.

## RESULTS

### Study characteristics

**FIGURE 1:**
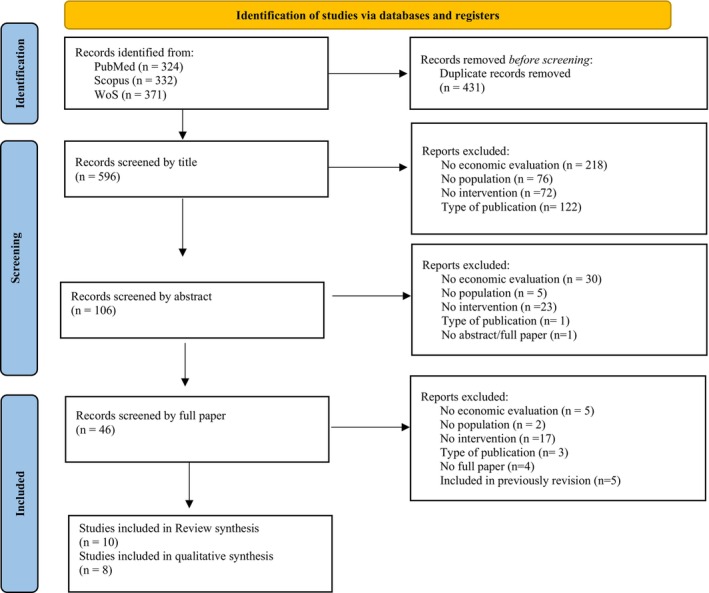
PRISMA‐ScR flow diagram illustrating the selection of economic papers.

The screening process (see Figure 1) involved 1027 articles from PubMed, Scopus, and Web of Science, among which 431 were identified as duplicates and consequently excluded. Screening of titles was conducted on 596 articles, leading to the removal of articles for the following reasons: 218 articles were excluded because they did not include an economic evaluation, 76 did not specify the target population, 72 did not involve any intervention relevant to the study objectives, and 122 due to their publication type. The remaining 106 articles were screened by abstract, resulting in 30 articles being excluded for not including an economic evaluation, five for not specifying the target population, 23 for not involving any relevant intervention, and one for publication type. Additionally, one article was eliminated for lacking both the abstract and the full paper.

Subsequently, 46 articles underwent full‐text assessment. Of these, 36 were excluded for the following reasons: no complete economic evaluation (*n* = 5), irrelevant population (*n* = 2), no intervention (*n* = 17), inappropriate type of publication (*n* = 3), no full paper available (*n* = 4), and already included in a previous review (*n* = 5). Ultimately, 10 articles met the initial inclusion criteria (Candio et al., 2022; Howe et al., 2022; Jacobs & Ellis, 2021; Kim, Rose, et al., 2024; Kim, Sookram, et al., 2024; Latimer et al., 2021; Liu et al., 2021; Palmer et al., 2020; Tam et al., 2019; van Mastrigt et al., 2020). Two of these studies (Kim, Rose, et al., 2024; Palmer et al., 2020) were excluded from the quantitative analysis as they were previous publications (e.g., clinical effectiveness) of the same interventions (Kim, Sookram, et al., 2024; Latimer et al., 2021). In conclusion, eight studies were included in the final analysis.

The eight studies included in this review, published between 2019 and 2024, are summarised in Tables 2 and 3. The included studies focus on interventions for stroke except one on TBI (Howe et al., 2022). No studies emerged about other forms of acquired brain injury. The geographical distribution of the studies included the United Kingdom (*n* = 1), Norway (*n* = 1), China (*n* = 1), the United States (*n* = 1), Canada (*n* = 1), the Netherlands (*n* = 1), Australia (*n* = 1) and across multiple European countries (*n* = 1). The interventions can be classified into two main categories: interventions for language disorders (Jacobs & Ellis, 2021; Kim, Rose, et al., 2024; Latimer et al., 2021; Liu et al., 2021) and the remaining four articles on general rehabilitation approaches, either focusing on a neuropsychological intervention (Howe et al., 2022) or including neuropsychological treatment within a more general rehabilitation framework (Candio et al., 2022; Tam et al., 2019; van Mastrigt et al., 2020).

**TABLE 2:** Summary of included studies.

Authors	Country	Study participants	Analysis, costs, health outcome measure, horizon	Interventions and comparator
**1. Rehabilitation of language disorders**				
Jacobs and Ellis (2021)	USA	Stroke patients	*Cost–benefit analysis* Health outcome measure: ASHA‐QCL was utilised to measure quality of communication life. Costs: marginal cost of treatment was calculated as the relationship between change in WAB‐R aphasia quotient (AQ) and the average cost per treatment. Horizon: N/A.	I: Telerehabilitation for aphasia C: No treatment
Kim, Rose, et al. (2024)	Australia	Stroke	*Cost‐effectiveness analysis* Health outcome measure: WAB‐R‐AQ (5.03‐point improvement from baseline to 26 weeks follow‐up) and the Stroke and Aphasia Quality of Life Scale‐39 (saqol‐39). Costs: Costs to patients, the health system and productivity costs. Horizon: Over a 26‐week period.	I: Usual care plus or VERSE therapy C: Usual care
Latimer et al. (2021)	UK	Stroke	*Cost‐utility analysis* Health outcome measure: Health utility estimated using EQ‐5D‐5L. Responses collected at baseline and follow‐ups (3, 6 and 12 months). Costs: Costs to patients, the health system and productivity costs. Horizon: life‐time.	I: Computerised word finding therapy plus usual care C: Usual care alone; usual care plus attention control
Liu et al. (2021)	China	Stroke	*Cost–utility analysis* Health outcome measure: raw short form 36 (SF‐36) health survey questionnaire data by parametric preference weights. Between the intervention and control groups from baseline to the end of the 4‐week intervention and 12‐week follow‐up period. Costs: The costs were evaluated from a societal perspective and included all expenses related to the interventions irrespective of who paid. Horizon: 12 weeks.	I: Speech and language therapy of aphasia C: Standard care
**2. General rehabilitation programs (comprehensive of neuropsychological interventions)**				
Howe et al. (2022)	Norway	Traumatic brain injury	*Cost‐utility analysis* Health outcome measure: Health utility estimated using EQ‐5D‐5L. Responses collected at baseline and follow‐ups (3, 6 and 12 months). Costs: Direct costs and societal costs (health and social care, informal care, productivity losses). Horizon: Over a 5‐year time.	I: Cognitive training intervention and individualised supported employment (CCT‐SE) C: Individualised outpatient treatment provided by a multidisciplinary team (TAU)
Tam et al. (2019)	Canada	Stroke	*Cost‐effectiveness analysis* Health outcome measure: difference between actual and projected LOS. Costs: FT costs (including costs of physician billings, medications, and various therapies) and the sum of projected costs of the ‘no FT’ alternative (usual care). Horizon: 12 weeks.	I: Fast‐track (FT) outpatient stroke rehabilitation program C: Usual care
van Mastrigt et al. (2020)	Netherlands	Stroke	*Cost‐utility analysis* Health outcome measure: Health utility estimated using EQ‐5D‐3L. Responses collected at baseline and follow‐ups (6 and 12 months). Costs: health care costs and non‐health care costs (intervention‐, patient‐ and family‐, and productivity costs). Horizon: life‐time.	I: Self‐management intervention (SMI) for stroke C: Control treatment (EDU)
Candio et al. (2022)	Europe	Stroke patients	*Cost‐utility analysis* Health outcome measure: Health utility estimated using EQ‐5D‐3L. Responses collected at 1 to 3, 6, 12, and 60 months. Costs: Direct costs and societal costs (health and social care, informal care, productivity losses). Horizon: Over a 5‐year time.	I: Home‐based rehabilitation C: Centre‐based rehabilitation

**TABLE 3:** Summary of included studies: Type and mode of intervention delivery.

	Authors	Type of intervention	Mode of intervention delivery
Rehabilitation of language disorders	Jacobs and Ellis (2021)	Neuropsychological intervention	Telerehabilitation treatment using the Language‐Oriented Treatment (LOT) delivered via Webex videoconferencing program. Each participant completed between 5 and 12 telehealth rehabilitation sessions of 45–60 min within a 6‐week time frame. Functional outcomes were evaluated by the NOMS (National Outcome Measurement System) and the ASHA‐QCL (American Speech Language and Hearing Association Quality of Communication Life Scale).
	Kim, Rose, et al. (2024)	Neuropsychological intervention	Usual Care Plus and VERSE (Very Early Rehabilitation in SpEech) therapies were commenced within 15 days of stroke and completed within 5 weeks of randomisation. These intervention arms received a total of 20 additional sessions (45–60 min, provided daily) of aphasia therapy, with *one arm receiving* additional usual ward‐based therapy (Usual Care Plus) and the other arm receiving a prescribed and structured intensive aphasia therapy program (the VERSE intervention). Functional outcomes were measured by the Boston Naming Test, the Stroke and Aphasia Quality of Life Scale‐39, and the Aphasia Depression Rating Scale.
	Latimer et al. (2021)	Neuropsychological intervention	Computerised therapy involved aphasia therapy software (StepByStep©) tailored to the participant's language impairment needs and personalised with 100 words relevant to the participant by a speech and language therapist. The participant was encouraged to practise word finding for 6 months daily. The intervention included monthly support from a speech and language therapist assistant or volunteer. The attention control group received puzzle books and monthly supportive telephone calls. Usual care (including speech and language therapy) continued to be provided to patients in all intervention groups so that the effectiveness of computerised therapy as an addition to usual care, could be assessed, rather than investigating computerised therapy as a replacement for usual care.
	Liu et al. (2021)	Neuropsychological intervention	In the SLT sessions, the intervention group received speech and language therapy immediately after scalp acupuncture, with needles remaining in place throughout. All participants completed the 4‐week intervention and 12‐week follow‐up. Standard care included identifying stroke type and risk factors, managing blood pressure, providing antiplatelet treatments and basic treatments for heart disease, and encouraging lifestyle changes such as weight control, exercise, smoking cessation, reduced alcohol consumption, low‐salt diet, increased fruit, and vegetable intake, and maintaining a positive mood. Functional outcome was measured with a Chinese version of the BDAE (Boston Diagnostic Aphasia Examination) and the CRRCAE (Chinese Rehabilitation Research Center Standard Aphasia Examination).
General rehabilitation programs	Howe et al. (2022)	Neuropsychological intervention	CCT was delivered in group sessions over 10 weeks by a clinical psychologist and a medical doctor, while SE was provided by three employment specialists over a 6‐month period. CCT sessions included psychoeducation and compensatory strategy training, with a vocational component based on the Individual Placement and Support model. SE focused on supporting participants in finding and maintaining employment. Therapists delivering CCT and SE collaborated closely to ensure the integration of strategies and techniques in workplace settings. TAU consisted of individualised assessment and treatment, along with participation in an education group focused on general TBI education and discussions of common challenges. TAU was provided for a maximum of 6 months, with individual contacts and participation in a 4‐week education group.
	Tam et al. (2019)	It includes neuropsychological rehabilitation as part of a general intervention	Introduced the fast‐track (FT) outpatient stroke rehabilitation program, wherein within 1 week of referral, patients could access outpatient rehabilitation services at an intensity like inpatient rehabilitation. FT's goal was to increase the rehabilitation service capacity by considering a fixed number of inpatient rehabilitation beds through earlier discharge or bypassing inpatient rehabilitation. Stroke patients were both ischaemic and haemorrhagic. The patients were treated by neuropsychologist and speech language pathologist. The total number of neuropsychological and speech language treatment was calculated.
	van Mastrigt et al. (2020)	It includes neuropsychological rehabilitation as part of a general intervention	The self‐management intervention (SMI) for stroke lasted 10 weeks, with weekly 2‐h sessions for the first 6 weeks and a 2‐h booster session in the tenth week. Conducted in outpatient facilities in the Netherlands, groups of four to eight participants (including up to four stroke patients and their partners) were led by a psychologist and a social worker. The training included proactive action planning on themes such as handling negative emotions, social support, societal participation, and less visible stroke consequences along with education on stroke consequences and peer support. The control treatment (EDU) also lasted 10 weeks, with three 1‐h sessions in the first 6 weeks and a 1‐h booster session in the tenth week. Delivered by a single rehabilitation professional, it covered themes like the brain and stroke, general stroke consequences, and stroke prevention. Patients were also educated by speech‐language therapists. Costs of both psychologists and speech‐language therapist were calculated.
	Candio et al. (2022)	It includes neuropsychological rehabilitation as part of a general intervention	Home‐based rehabilitation included physiotherapy, occupational therapy, and speech therapy at home. Compared to centre‐based rehabilitation (CB) with conventional hospital‐based care (inpatient and outpatient). Each session of Speech‐Language therapy consisted of a 20‐min treatment with a therapist. The mean costs for both home‐based and centre‐based treatments were recorded.

Key outcomes used to evaluate the interventions vary across the studies included. They included Health utility estimated using EQ‐5D‐3L or EQ‐5D‐5L, as documented in the studies by Latimer et al. (2021), Howe et al. (2022), van Mastrigt et al. (2020), and Candio et al. (2022). Additionally, measures of quality of life were assessed using different questionnaires (Kim, Rose, et al., 2024; Liu et al., 2021). Jacobs and Ellis (2021) took into consideration the quality of communication. Furthermore, Tam et al. (2019) considered the length of hospital stays as the crucial indicator of intervention effectiveness. Costs considered in the analyses encompassed direct health care costs, societal costs, informal care, and productivity losses. Whenever possible, in the description of the studies with general rehabilitation procedures, the portion of costs ascribable to the neuropsychological intervention is reported. The studies varied in their time horizons, ranging from short‐term evaluations over weeks to long‐term evaluations spanning several years.

Table 3 presents the modalities of intervention delivery of the included studies. Note that the settings in which the interventions were implemented vary. Jacobs and Ellis (2021), Latimer et al. (2021), and Candio et al. (2022) evaluated home‐based care programs. In the remaining studies, interventions were delivered in an outpatient setting. These interventions were compared against standard care, centre‐based rehabilitation, individualised outpatient treatment, no treatment, or alternative therapies.

### Intervention characteristics

Four studies (Jacobs & Ellis, 2021; Kim, Rose, et al., 2024; Latimer et al., 2021; Liu et al., 2021) aimed at treating language disorders, particularly in patients with stroke and fluent types of aphasia. These interventions included Language‐Oriented Treatment, intensive therapy for anomic and conduction aphasia, computerised word‐finding therapy, and language therapy accompanied by scalp acupuncture (see Table 3).

Conversely, the other studies featured general rehabilitation programs, including different neuropsychological interventions to varying extents. For example, in Howe et al.'s (2022) study, the treatment mostly involved a neuropsychological intervention featuring psychoeducational and compensatory strategy training. The other studies encapsulated neuropsychological interventions (such as speech‐language training delivered by neuropsychologists and speech‐language for stroke patients; Tam et al., 2019) within general rehabilitation programs (see Table 3).

### Perspective

The studies included in this analysis adopt various perspectives (see Table 4), each offering unique insights into the cost‐effectiveness of interventions for stroke and traumatic brain injury (TBI) patients. Particularly, the focus is on the National Health Service (NHS) and Personal Social Services, indicating a broad perspective encompassing health care and social care elements (see Table 4).

**TABLE 4:** Summary of included studies: Results of cost analyses conducted.

Study	Study perspective	Type and source(s) of cost data	Mean costs and health effects (95% CI)	Difference in incremental cost‐effectiveness ratio	Claimed findings/authors' conclusions
**Rehabilitation of language disorders**					
Jacobs and Ellis (2021)	Single health care Payer prospective	To calculate the costs of aphasia treatment the total billed cost of treatment was calculated as the total cost of all treatment sessions attended. *Source*: Medicare sources tariff (actualised to 2021).	N/A	The monetary equivalent in the patient's improved QCL was between $1,790.39 (€1.580,83) to $3,912,54 (€3.454,58), far exceeding the financial cost of treatment.	As tele‐practice treatments for aphasia are becoming more widely used, information related to the cost of care and cost‐effectiveness of treatments provided is needed.
Kim, Rose, et al. (2024)^a^	NHS and Personal Social Services	Costs involved in the provision of aphasia treatments during the hospital stay, costs of health care resources used, and other costs thought to be influenced by the type of aphasia treatment received incurred beyond the health sector (such as the time costs associated with informal care provided by family members and the productivity gains/losses and out‐of‐pocket costs) were considered. *Source*: Australian sources tariff (actualised to 2017).	Median costs per person were Usual Care $23,322 (Q1 5367, Q3 52,669)	ICER: N/A. Usual Care Plus was inferior (i.e., more costly and less effective) in 64% of iterations, and, in 18%, less costly and less effective compared to Usual Care. VERSE was inferior in 65% of samples and less costly and less effective in 12% compared to Usual Care.	There was no evidence that intensive aphasia therapy was cost‐effective when provided within the first 5 weeks after stroke, with no evidence that this was affected by aphasia severity or the amount of therapy provided. Speech therapy continued to be provided within the acute and sub‐acute hospital setting as part of standard rehabilitation services, but there remained limited evidence about the best type, timing, and intensity of therapy for patients with aphasia.
			Usual Care Plus $26,923 (Q1 7303, Q3 76,174)		
			VERSE $31,143 (Q1 7001, Q3 62,390)		
Latimer et al. (2021)	NHS and Personal Social Services	Costs for computerised therapy encompassed hardware such as computers and headsets (for loaned equipment), StepByStep© software, and the time invested by speech and language therapists and assistants in delivering and training for the intervention, along with associated travel expenses. Attention control costs comprised puzzle books and staff time for monthly participant phone calls. As usual care was uniform across intervention groups, costs linked to usual care were omitted from the economic assessment. *Source*: UK *unit costs of health and social care* (actualised to 2019).	Comparator: usual care alone C: £732.73 (€858,09) vs. £0.00 Q: 4.2164 vs. 4.1992	In the base‐case analysis, computerised therapy plus usual care yielded an ICER of £42,686 (€49.989,1) per QALY gained compared to usual care alone. The incremental cost per patient was £732.73 (€858,093) with a 95% credible interval of £674.23–£798.05 (€789,584– €934.588), and the incremental QALY gain per patient ranged from .02 to .10. Compared to attention control plus usual care, computerised therapy plus usual care had an ICER of £40,164 (€47.035,7) per QALY gained. Attention control plus usual care was economically inferior, being more expensive and yielding fewer QALYs compared to usual care alone. Based on a threshold of £30,000 (€35.132,7) per QALY gained, the probability of computerised therapy plus usual care being the most cost‐effective option was 32%, while it was 45% for usual care alone and 22% for attention control plus usual care.	Computerised word‐finding therapy represents a low‐cost add‐on to usual care, but QALY gains and estimates of cost‐effectiveness are uncertain. Computerised therapy is more likely to be cost‐effective for people with mild or moderate, as opposed to severe, word‐finding difficulties.
			Incremental cost £732.73 (£674.23–£798.05) €858,09 (€789,58–€934,59)		
			Incremental QALYs .0172 (−.05 to .10)		
			Comparator: attention control plus usual care C: £732.73 (€858,09) vs. £38.14 (€44,47) Q: 4.2164 vs. 4.1991		
			Incremental cost £694.59 (£636.46–£760.09) €813,427 (€745,35–€890,13)		
			Incremental QALYs .0173 (−.05 to .10)		
Liu et al. (2021)^a^	NHS and Personal Social Services	Direct individual costs were incurred from the duration of hospitalisation, visits to the emergency department, general medical treatments, visits to a specialised physician and nursing care. Direct non‐medical costs included the carer's transportation expenses to and from treatment sessions. Regarding indirect costs from a societal perspective, the per‐day cost of sick leave from work during rehabilitation (both participants and their family members) was assessed using a human capital approach ((China's per capita Gross Domestic Product (GDP) in 2015 ÷ 365 days) × the number of days sick from work). *Source*: China tariff 2014 (actualised to 2016).	Average direct medical costs per participant: Intervention group: ¥4780.70 (€610,84) Control group: ¥6059.49 (€774,23)	The ratio of incremental cost per QALY was ¥ −39,378.67 (equivalent to € −5031.45) after the 12‐week follow‐up. Considering the societal perspective, if the willingness‐to‐pay threshold is established at ¥50,696 (equivalent to €6,477.48) GDP per capita in 2015, there is a 56.4% likelihood that the intervention group will be deemed cost‐effective.	The results revealed negative ICERs, suggesting the cost‐effectiveness of the intervention approach, thus favouring it as the preferred option. Specifically, the augmentation of scalp acupuncture to speech and language therapy demonstrated cost‐effectiveness within Chinese communities. However, given the potential variation in acupuncture costs across different regions, especially compared to other countries, it's imperative to interpret these findings cautiously. Further validation in diverse populations is warranted to ascertain its broader applicability.
			Total costs: Intervention: ¥29,376.59 (€3.753,48) Control: ¥31,739.31 (€4055.36)		
			Direct non‐medical costs: Intervention group: ¥2391.55 (€305.57) Control group: ¥4382.86 (€560.00)		
			QALYs: Intervention group: .62 ± .10 Control group: .55 ± .10 Mean difference in QALYs: .07 (95% CI: .06–.08)		
**General rehabilitation programs (comprehensive of neuropsychological interventions)**					
Howe et al. (2022)	NHS and Personal Social Services	Primary Care Cost: Number of visits to a general practitioner, physical therapist, chiropractor, and contract specialists. Contract Specialists Cost: Includes expenses for consultations with dentists, neurologists, ophthalmologists, orthoptists/opticians, otorhinolaryngologists, and psychologists. Also covers out‐of‐pocket services such as naprapathy and osteopathy. Informal Care: Quantified by the hours of assistance received per week from friends or family. Production Loss: Represents the financial impact of reduced productivity or work absence due to injury or illness. *Source*: Norwegian tariff (actualised to 2019).	Health care perspective: CCT‐SE C: €3.006 Q: .111 TAU: C: €2.025 Q: .071	€19.260 (€22.871,25) per QALY from a health care perspective; ‐€109.900 (‐€130.506) per QALY from a societal perspective.	CCT‐SE is a cost‐effective alternative from a societal perspective, but not from a health care perspective.
			**Incremental values (95% CI)** C: ‐€979 (−159 to 1.877) Q: .042 (−.036 to .138) Societal prospective CCT‐SE C: €36.296, Q: .107 TAU: C: €37.615, Q: .096		
			**Incremental values (95% CI)** C: ‐€1.319 (‐€19.643 to €11.807) Q: .012 (−.085 to .088)		
Tam et al. (2019)^a^	Single health care payer perspective	Publicly funded health care costs included family physician billings, physiatrist billings, TRI inpatient rehabilitation bed per diem cost, physiotherapy services, occupational therapy (OT), speech‐language pathology (SLP), social work and neuropsychology. Medication costs. Hospital costs for the ‘no FT’ alternative were estimated using target LOS generated by the RPG assignment multiplied by average daily costs. UHN finance calculated the cost for one inpatient stroke rehabilitation day. Clinical billing patterns were collected through interviews with the family physician and physiatrist working in the stroke rehabilitation unit and FT. *Source*: Canadian tariff 2016 (actualised to 2016).	Incremental costs derived for TRI and TWH were $2033 (€1.432,36) (95% CI: $1509 (€1.063,18)–2558 (€1.802,26)) and $520 (95% CI: $276–765), respectively	ICER for TRI patients was $404 (€284,64) (95% CI: $270–620 (€190,23–436,83)) per inpatient day saved. The estimated ICER for TWH patients was $37 (€26) (95% CI: $20–55 (14.09–38.75)) per inpatient day saved.	The FT program is cost‐effective, as it focuses resources directly on rehabilitation therapy for patients and avoids custodial care costs.
			Incremental effects for TRI and TWH were 5.04 and 14 inpatient days saved, respectively		
van Mastrigt et al. (2020)^a^	NHS and Personal Social Services	For the Self‐Management Intervention (SMI), costs encompassed various elements such as the wages of medical professionals involved, potential day training for these professionals, and workbooks for both professionals and patients. Similarly, for the Educational Intervention (EDU), costs included medical professionals' wages, potential education training sessions, and provision of workbooks. Health care costs were computed using the Dutch Manual for Costing, except for medication costs. This covered various health care services like GP and specialist consultations, medication, and home care. Prescription drug costs were based on dosage prices in the Netherlands, while medical aids were calculated per user category. Patient and family costs covered informal care and travel expenses, with informal care valued based on professional caregiver wages. Travel costs were determined by multiplying distance with standard price weights and adjusting for public transport and parking. Productivity costs were evaluated using the human capital approach, factoring in sick days and labour costs across different age categories. *Source*: Netherlands, Dutch Care Institute and Dutch Manual for Costing tariff (actualised to 2012).	The average total societal costs between Self‐Management Intervention (SMI) and Educational Intervention (EDU) were not significantly different, with SMI costing €17,333 compared to €15,520 for EDU, showing a 95% confidence interval (CI) of €‐3380 to €7099. However, there were notable variations in specific cost categories	This slightly higher QALY for SMI resulted in an ICUR of €44,688. Table 4 shows that 73% of the bootstrapped pairs were located in the NE quadrant (higher effects and higher costs) of the CE plane, and 23% were in the SE quadrant (higher effects and lower costs). Assuming a willingness to pay (WTP) threshold of €50,000 per QALY, the probability that SMI will be cost‐effective is 52%.	SMI is probably not a cost‐effective alternative in comparison with EDU. Based on the current results, the value of implementing SMI for a stroke population is debatable. We recommend further exploration of the potential cost‐effectiveness of stroke‐specific self‐management interventions, focusing on different underlying mechanisms and using different control treatments.
			Total health care costs were slightly higher for SMI at €6138 compared to €5899 for EDU, with a 95% CI of €‐2352 to €2685. Conversely, total non‐health care costs were higher for SMI at €11,320 compared to €9507 for EDU, with a 95% CI of €‐1923 to €5769		
			Three cost categories exhibited significant differences between the interventions. Activity therapy costs were substantially higher for SMI at €276 compared to €65 for EDU (95% CI €24 to €452). Conversely, costs for tools and home adjustments were notably higher for EDU at €231 and €192, respectively, compared to SMI, with 95% CI (€‐272, €‐3)		
			Although not statistically significant, productivity costs were notably larger for SMI at €5392 compared to €4187 for EDU. Intervention costs were also higher for SMI at €764 compared to €222 for EDU		
			Furthermore, the Quality‐Adjusted Life Years (QALY) for baseline utility differences were .715 for SMI and .672 for EDU		
Candio et al. (2022)	NHS and Personal Social Services	Treatment costs: Physiotherapy; Occupational therapy; Speech therapy. Health and social care resource: Hospital stay and day cases (inpatient costs), outpatient visits, accident, and emergency (A&E) visits and nursing/residential care (for patients aged at least 65 years old). Informal care costs. Productivity losses: loss of productivity was calculated in terms of mortality and morbidity in stroke patients under the age of 65 years. *Source*: UK *unit costs of health and social care* 2008 (actualised to 2017).	Home‐based rehabilitation resulted in 5‐year cost savings compared to CB‐rehabilitation (€43.8 billion vs. €44.1 billion to society)	Cost savings of €237 (€287.04) million (95% CI: −237 to 1.764) in health and social care costs. Cost savings of €352 (€426.33) million (95% CI: −340 to 2.237) in societal costs.	Shift to home‐based stroke rehab is likely cost‐effective across Europe.
			Home‐based rehabilitation in Europe generated an additional 59.211 LYs (95% CI: −1558 to 109.975) and 61,888 QALYs (3.609–118.679)		

^a^ Economic evaluation was reported according to the Consolidated Health Economic Evaluation Reporting Standards checklist (Husereau et al., 2013).

Studies by Candio et al. (2022), Howe et al. (2022), Kim, Rose, et al. (2024), Latimer et al. (2021), Liu et al. (2021), and van Mastrigt et al. (2020) investigate the impact of interventions within the NHS and Personal Social Services, emphasising the intersection of health and social care, providing comprehensive insights into the efficacy and impact of various health care interventions.

In contrast, Jacobs and Ellis (2021) and Tam et al. (2019) provide a perspective by adopting a Single health care Payer prospective approach, focusing on the implications of interventions within a single health care payer system that includes only the costs and impacts on the specific payer predominantly from the health care sector, without considering the social implications of interventions and comparators.

Altogether, these diverse perspectives enrich our understanding of the cost‐effectiveness of interventions for stroke and TBI patients, highlighting the importance of considering various stakeholders and societal factors in economic evaluations.

### Type and source(s) of costs

Each study reports all costs associated with each intervention and comparators, using diverse data sources and methodologies. Candio et al. (2022) and Kim, Rose, et al. (2024) scrutinised treatment costs and health and social care resource utilisation. Candio et al. (2022) included informal care costs and productivity losses, relying on UK unit costs of health and social care tariffs. Kim, Rose, et al. (2024) expanded their scope to include costs beyond the health sector, using Australian tariffs. Jacobs and Ellis (2021) and Latimer et al. (2021) focused on specific treatment costs, while Jacobs and Ellis (2021) analysed total billed costs of aphasia treatment from health care billing records, and Latimer et al. (2021) compared computerised therapy, attention control, and usual care costs, using UK unit costs of health and social care tariffs.

Howe et al. (2022) and Tam et al. (2019) delved into primary care costs. Howe et al. (2022) added an analysis of contract specialist costs, informal care, and production losses using Norwegian tariff data, while Tam et al. (2019) focused both on specialist costs (specifically also on speech‐language pathologists and neuropsychologists) and publicly funded health care and medication costs with Canadian tariffs. Liu et al. (2021) and van Mastrigt et al. (2020) took a more holistic approach by examining direct individual, non‐medical, and indirect societal costs. Liu et al. (2021) used China tariff data, whereas van Mastrigt et al. (2020) drew upon data from the Dutch Care Institute and the Dutch Manual for Costing to analyse health care, non‐health care, patient and family, and productivity costs.

These varied approaches provide a rich understanding of the economic implications of the interventions studied, reflecting the multifaceted nature of health care economics.

### Cost‐effectiveness results

The economic analyses conducted comprised two cost‐effectiveness (Kim, Sookram, et al., 2024; Tam et al., 2019), five cost‐utility (Candio et al., 2022; Howe et al., 2022; Latimer et al., 2021; Liu et al., 2021; van Mastrigt et al., 2020), and one cost–benefit (Jacobs & Ellis, 2021) analyses to evaluate the impact of the target interventions. Howe et al. (2022), the only study evaluating the rehabilitation of TBI patients, provided a cost‐utility analysis.

Among the eight studies analysed, it is notable that five of them (Kim, Sookram, et al., 2024; Latimer et al., 2021; Liu et al., 2021; Tam et al., 2019; van Mastrigt et al., 2020) adhered to the reporting criteria laid out in the Consolidated Health Economic Evaluation Reporting Standards (CHEERS), as outlined by Husereau et al. (2013). This adherence signifies transparency and methodological rigour in presenting the economic evaluations of the interventions. By aligning with the CHEERS guidelines, these studies provide a comprehensive and standardised framework for reporting the key elements of their cost‐effectiveness analyses, ensuring clarity, transparency and reproducibility, researchers, policymakers, and health care practitioners to scrutinise, validate, and build upon the findings presented in these studies.

Howe et al. (2022) is the only economic evaluation of an intervention relative to traumatic brain injury. The authors conducted a cost‐utility analysis comparing cognitive training intervention and individualised supported employment (CCT‐SE) with individualised outpatient treatment provided by a multidisciplinary team (treatment as usual). Their findings indicated that CCT‐SE was cost‐effective from a societal perspective, with an incremental cost‐utility ratio (ICUR) of €22,871 per QALY gained. This suggests that although CCT‐SE is associated with higher costs, it also leads to greater effectiveness in terms of health outcomes, making it a favourable option from a societal standpoint.

The remaining seven papers are related to patients after stroke. Four (Jacobs & Ellis, 2021; Kim, Rose, et al., 2024; Latimer et al., 2021; Liu et al., 2021) focused on the cost‐analysis of interventions for language disorders. Jacobs and Ellis (2021) conducted a cost–benefit analysis of telerehabilitation compared to a no‐treatment condition. These authors found positive outcomes for improved quality of communication life, suggesting the potential economic benefits of tele‐practice treatments for aphasia with the monetary equivalent in patient's improved QCL was between €1.580,83 to €3.454,58, far exceeding the financial cost of treatment. Latimer et al. (2021) evaluated the cost‐utility of computerised word‐finding therapy plus usual care compared to usual care alone. Their analysis revealed an ICER of €49.989,1 per QALY gained for adding computerised training, suggesting that it represents a low‐cost enhancement to standard care. However, the cost‐effectiveness remained uncertain, particularly concerning the severity of word‐finding difficulties. Kim, Rose, et al. (2024) performed a cost‐effectiveness analysis. However, they did not report the ICER but only the probability of the treatments being cost‐effective. Their analysis showed that *Usual Care Plus* dominated (i.e., more costly, and less effective) in 64% of cases compared to Usual Care. Similarly, VERSE dominated in 65% of cases and was less costly, and less effective in 12% compared to Usual Care. The last study on language disorder interventions is that by Liu et al. (2021). They conducted a cost‐utility analysis within the NHS and Personal Social Services perspective, assessing the cost‐utility of scalp acupuncture augmentation to speech and language therapy. They reported a negative ICER (i.e., less costly and more effective), indicating the cost‐efficacy of the intervention approach, particularly within Chinese communities.

The remaining three studies focused on the economic analysis of general rehabilitation programs, comprehensive of neuropsychological treatments for post‐stroke patients, providing insights into different intervention strategies (Candio et al., 2022; Tam et al., 2019; van Mastrigt et al., 2020). Tam et al. (2019) focused on a single health care payer perspective and assessed the cost‐effectiveness of a fast‐track outpatient stroke rehabilitation program. The reported ICERs of €284.64 per QALY and €26 per inpatient day saved for the different components of the program indicate that the rapid outpatient program offers cost‐effective benefits over usual care, particularly in reducing inpatient days and associated costs. Opposite results emerge from van Mastrigt et al. (2020), who conducted a cost‐utility analysis comparing self‐management intervention (SMI) for stroke with control treatment (EDU). They reported an ICUR of 44,688 per QALY gained for SMI, which may offer some benefits but is probably not a cost‐effective alternative to the control treatment because it does not provide sufficient improvements in quality‐adjusted life years relative to its costs. The last included paper is Candio et al. (2022) which performed a cost‐utility analysis within the NHS and Personal Social Services perspective, comparing home‐based rehabilitation with centre‐based rehabilitation around different European countries. Their study revealed significant cost savings of €287 million over 5 years with home‐based rehabilitation compared to centre‐based rehabilitation. Home‐based rehabilitation could produce an improvement in terms of life‐years (LYs) and quality‐adjusted life years (QALYs). Additionally, the intervention resulted in €426 million in health and social care cost savings, underscoring substantial economic and health benefits.

## DISCUSSION

This review aimed to highlight the new economic evidence published after 2019 on the cost‐effectiveness of neuropsychological rehabilitation interventions in individuals with ABI after stroke or TBI. As in the review by Stolwyk et al. (2021), we identified eight studies with a cost‐effectiveness or cost‐utility design. However, the previous review covered a much broader time frame (1995–May 2019) than the present one (2019–May 2024). The same number of articles in a much shorter time frame suggests a growing recognition of the importance of evaluating the economic impact of neuropsychological rehabilitation interventions. Indeed, the change in social demographics in economically advanced countries has led to greater attention being paid to the economic aspects of health care. Due to a progressively ageing population, new approaches to neuropsychological rehabilitation have been explored to face economic issues. For example, Jacobs and Ellis (2021) found economic benefits for telerehabilitation of patients with severe aphasia, indicating that they exceeded the treatment cost. Indeed, neuropsychological telerehabilitation is a critical challenge for future developments because of its great potential to reduce costs and favour patient's participation in rehabilitation interventions (Cacciante et al., 2022).

We noted that the included studies fall into two general domains. Some studies focused on a specific and severely debilitating symptom following the brain injury that is the presence of communication difficulties due to language impairments. Language disorders represent a key disturbance after a brain lesion, indeed one of the very first areas of research to trigger neuropsychological analysis. The reviewed evidence provides some support for the cost‐effectiveness of rehabilitation treatments focused on communication disorders. Jacobs and Ellis (2021) also found economic benefits for aphasia telerehabilitation, indicating that the economic benefits exceed the treatment costs. Kim, Rose, et al. (2024) showed that additional sessions of aphasia therapy were generally less cost‐effective than usual care. Despite some uncertainty, Latimer et al. (2021) found potential cost‐effectiveness for computerised word‐finding therapy. Liu et al. (2021) indicated that adding scalp acupuncture to speech therapy was cost‐effective, particularly in Chinese communities.

Language disorders represent a key disturbance after a brain lesion, indeed one of the very first areas to trigger neuropsychological analysis of cognitive impairments. Research on the neuropsychological rehabilitation of language disorders also has a long‐lasting tradition. Descriptive reviews have confirmed the efficacy of language rehabilitation training (e.g., Basso et al., 2013). More recent systematic reviews and meta‐analyses have maintained the general idea that language rehabilitation is more effective than usual care but have also underscored the importance of further studies with high‐quality methodological designs (Brady et al., 2016; Shrubsole et al., 2017). In this, vein, some authors have emphasised the importance of considering the large individual variability in response to rehabilitation (Doogan et al., 2018). Furthermore, recent evidence indicates that transcranial brain stimulation may prove beneficial to aphasia recovery (Bucur & Papagno, 2019), possibly potentiating the effect of behavioural rehabilitation (Marangolo, 2020). Finally, another important area in which additional knowledge would be important (particularly because of its organisational implications) is understanding the efficacy of telerehabilitation of language disorders (Cacciante et al., 2021). The present findings on the cost‐effectiveness of language rehabilitation present a generally similar picture. Thus, there is some evidence that rehabilitation training focused on communication disorders is cost‐effective; however, the picture is still incomplete, and more evidence is needed to specify the most effective forms of linguistic treatment. A key message from the present analysis is that the quest for methodologically valid studies on language rehabilitation, should probably jointly consider clinical effectiveness and cost‐effectiveness as key targets of their analysis.

Other studies examined different forms of neuropsychological interventions delivered as part of general rehabilitation programs. Three studies focused on stroke patients. Tam et al. (2019) highlighted the cost‐effectiveness of a fast‐track outpatient rehabilitation program. By contrast, van Mastrigt et al. (2020) suggested that a self‐management intervention was probably not cost‐effective compared to a control treatment. In the only study on TBI patients, Howe et al. (2022) demonstrated that neuropsychological rehabilitation including cognitive training with supported employment was cost‐effective (less expensive and more effective) compared to usual treatment, although only from a societal (not health) perspective. Finally, Candio et al. (2022) carried out a complex analysis based on data from a population‐based cohort and previous studies, to develop a state‐transition cohort model to simulate the impact of rehabilitation across several European countries. They reported that home‐based rehabilitation was expected to be cost‐effective (less expensive and more effective) in most European countries. Overall, the scope and breadth of these studies are as variable as the forms of neuropsychological interventions they refer to. Furthermore, only one study concerned patients with TBI, a particularly critical condition given the frequently young age of the affected individuals. These factors make it difficult to draw general conclusions from this dataset, even though there are several indications that neuropsychological intervention may prove cost‐effective, thus supporting the interest in further pursuing this type of research.

Comparing the time frame of this review with that of Stolwyk et al. (2021), the number of studies included in this review is significantly higher. This increase highlights a growing recognition of the need for economic evaluations in stroke and brain injury rehabilitation. Given the expected significant increase in the incidence of these conditions, the full implementation of economic analyses is critical to provide policymakers with concrete evidence. This approach will aid in the development and adoption of cost‐effective interventions, thereby optimising resource allocation and improving patient outcomes.

We suggest that future research on the economic evaluation in neuropsychological rehabilitation should combine tools, such as Quality‐Adjusted Life Years (QALYs), with specific clinical outcome measures to provide a robust assessment of both economic and therapeutic benefits. Additionally, qualitative evaluations should be integrated by collecting data through patient and clinician interviews and focus groups to capture nuanced perspectives on treatment impact. It is also crucial to use real‐world data from clinical registries and administrative databases to reflect the actual costs and benefits in practical settings. Using this comprehensive approach, would be possible not only to compare the costs of different interventions but also to assess their efficacy, providing policymakers with the necessary information to make informed decisions, ultimately leading to improvements in neuropsychological rehabilitation practices.

### Strengths and limitations

For robust economic analyses, it is imperative to have appropriate follow‐up data to assess the sustainability of gains in outcome measures and cost offsets over the long term. As underscored by Stolwyk et al. (2021), making assumptions about long‐term costs and benefits without sufficient follow‐up data can compromise the robustness of the results. More than half of the studies with a cost‐effectiveness design demonstrated a strong adherence to the reporting criteria outlined in the CHEERS guidelines (Husereau et al., 2013). By following these guidelines, these studies establish a robust and standardised framework for reporting critical aspects of their cost‐effectiveness analyses.

Since we used the same search criteria as Stolwyk et al. (2021), the limitations are similar. The first one concerns the search query because we recognise that the two reviews are not exhaustive as they aimed to conduct a scoping review focusing on neuropsychological rehabilitation. This decision may have resulted in the omission of some potentially relevant studies with cost‐effectiveness data from other rehabilitation disciplines like occupational therapy. Additionally, studies that did not mention economic‐relevant terms in titles, abstracts, or keywords might have been not emerged from the search.

Furthermore, we did not include searches of grey literature, focusing instead on peer‐reviewed literature to evaluate economic and intervention methodologies. In future work, it will be useful to include these analyses from grey literature sources about economic evaluations.

Finally, due to the diversity in study methods, samples, interventions, and cost analyses, we did not conduct a meta‐analysis.

## CONCLUSIONS

This study provides a review of the cost‐effectiveness of neuropsychological interventions for stroke and brain injury in adults across various health care settings after 2019. Through various analyses, such as cost‐utility and cost–benefit analyses, the assessment provides information on the economic viability of these interventions. The inclusion of multiple perspectives, including societal and health system perspectives, highlights the comprehensive approach taken in the literature to assess the overall value of health care interventions. However, the heterogeneity of the findings underscores the need for further research and ongoing evaluation to identify the most cost‐effective approaches for treating patients with stroke and TBI, particularly with longer follow‐up periods.

As highlighted by Stolwyk et al. (2021), research in this field is expanding, as evidenced by the increased number of studies over the 5 years between the two reviews. In their analysis, these authors noted that most studies focussed on multidisciplinary rehabilitation programs with limited possibility to delineate neuropsychological and cognitive interventions; indeed, they did not find studies examining the cost‐effectiveness of specific cognitive interventions. Similarly, in the present review, about half of the studies concerned multidisciplinary interventions, with neuropsychological and cognitive interventions blended within a more general rehabilitation program. However, we also found a significant emergence of studies focusing on the cost‐effectiveness of language rehabilitation, a type of training with a long‐lasting tradition as well as a currently active field of research. Notably, other critical neuropsychological symptoms have not yet received the same attention. For, example, there is by now a consolidated tradition of studies on the rehabilitation of spatial neglect, a deficit with widespread influence on the quality of life of the affected patients (for recent reviews, see Liu et al., 2019; Meidian et al., 2022) but we could not trace any study on the cost‐effectiveness of this type of intervention, and this remains as a potentially interesting area of future research.

Moreover, the growing interest in costs and sustainability in neuropsychological rehabilitation has induced clinicians to explore new modalities to deliver rehabilitation treatments compared to conventional treatments one to one in person. Telerehabilitation has been evaluated as part of the present analysis and seems sustainable, even though its effectiveness is still unclear.

In conclusion, this review, which builds on the original work of Stolwyk et al. (2021), documents the recent growth of studies on the cost‐effectiveness analysis of neuropsychological interventions, particularly concerning interventions in the language domain. The economic analysis of these studies emphasises the relative homogeneity in cost‐effectiveness outcomes across different interventions. It also underscores the importance of further assessments of the cost‐effectiveness of rehabilitation interventions to optimise health care practices and policy decisions. Prioritising cost‐effective treatments can enhance patient care and resource allocation, ultimately leading to improved health outcomes and sustainability in health care systems.

## AUTHOR CONTRIBUTIONS

**Mauro Mancuso:** Conceptualization; data curation; investigation; formal analysis; project administration; validation; resources; writing – review and editing; writing – original draft; visualization. **Ilaria Valentini:** Conceptualization; data curation; investigation; formal analysis; validation; resources; visualization; writing – review and editing; writing – original draft. **Michele Basile:** Conceptualization; data curation; investigation; formal analysis; validation; resources; visualization; writing – review and editing; writing – original draft. **Audrey Bowen:** Conceptualization; writing – review and editing. **Helena Fordell:** Conceptualization; writing – review and editing. **Roberta Laurita:** Resources; writing – review and editing; writing – original draft. **Marika C. Möller:** Writing – review and editing; conceptualization. **Lindy J. Williams:** Conceptualization; writing – review and editing. **Pierluigi Zoccolotti:** Conceptualization; data curation; formal analysis; investigation; resources; supervision; validation; visualization; writing – review and editing; writing – original draft.

## CONFLICT OF INTEREST STATEMENT

Prof. Audrey Bowen is a co‐author of one of the articles that was selected as part of the present scoping review (Latimer et al., 2021). The other authors have no conflicts of interest to declare.

## Supporting information


Table S1

## Data Availability

The data that support the findings of this study are available from the corresponding author upon reasonable request.
